# Maternal Iron Status in Pregnancy and Child Health Outcomes after Birth: A Systematic Review and Meta-Analysis

**DOI:** 10.3390/nu13072221

**Published:** 2021-06-28

**Authors:** Hugo G. Quezada-Pinedo, Florian Cassel, Liesbeth Duijts, Martina U. Muckenthaler, Max Gassmann, Vincent W. V. Jaddoe, Irwin K. M. Reiss, Marijn J. Vermeulen

**Affiliations:** 1 The Generation R Study Group, Erasmus MC-Sophia, University Medical Center, P.O. Box 2060, 3000 CB Rotterdam, The Netherlands; h.quezadapinedo@erasmusmc.nl (H.G.Q.-P.); v.jaddoe@erasmusmc.nl (V.W.V.J.); i.reiss@erasmusmc.nl (I.K.M.R.); 2 Department of Pediatrics, Erasmus MC-Sophia, University Medical Center, P.O. Box 2060, 3000 CB Rotterdam, The Netherlands; 3 Department of Pediatrics, Division of Neonatology, Erasmus MC-Sophia, University Medical Center, P.O. Box 2060, 3000 CB Rotterdam, The Netherlands; f.cassel@erasmusmc.nl (F.C.); l.duijts@erasmusmc.nl (L.D.); 4 Department of Pediatrics, Division of Respiratory Medicine and Allergology, Erasmus MC-Sophia, University Medical Center, P.O. Box 2060, 3000 CB Rotterdam, The Netherlands; 5 Molecular Medicine Partnership Unit, University Hospital Heidelberg, D-69120 Heidelberg, Germany; Martina.Muckenthaler@med.uni-heidelberg.de; 6 Institute of Veterinary Physiology, Vetsuisse Faculty, University of Zurich, CH-8057 Zurich, Switzerland; maxg@access.uzh.ch; 7 Zurich Center for Integrative, Human Physiology, University of Zurich, CH-8057 Zurich, Switzerland; 8 School of Medicine, Universidad Peruana Cayetano Heredia, Lima 15102, Peru

**Keywords:** nutrients, fetal programming, long term outcomes, gestation, offspring

## Abstract

In pregnancy, iron deficiency and iron overload increase the risk for adverse pregnancy outcomes, but the effects of maternal iron status on long-term child health are poorly understood. The aim of the study was to systematically review and analyze the literature on maternal iron status in pregnancy and long-term outcomes in the offspring after birth. We report a systematic review on maternal iron status during pregnancy in relation to child health outcomes after birth, from database inception until 21 January 2021, with methodological quality rating (Newcastle-Ottawa tool) and random-effect meta-analysis. (PROSPERO, CRD42020162202). The search identified 8139 studies, of which 44 were included, describing 12,7849 mother–child pairs. Heterogeneity amongst the studies was strong. Methodological quality was predominantly moderate to high. Iron status was measured usually late in pregnancy. The majority of studies compared categories based on maternal ferritin, however, definitions of iron deficiency differed across studies. The follow-up period was predominantly limited to infancy. Fifteen studies reported outcomes on child iron status or hemoglobin, 20 on neurodevelopmental outcomes, and the remainder on a variety of other outcomes. In half of the studies, low maternal iron status or iron deficiency was associated with adverse outcomes in children. Meta-analyses showed an association of maternal ferritin with child soluble transferrin receptor concentrations, though child ferritin, transferrin saturation, or hemoglobin values showed no consistent association. Studies on maternal iron status above normal, or iron excess, suggest deleterious effects on infant growth, cognition, and childhood Type 1 diabetes. Maternal iron status in pregnancy was not consistently associated with child iron status after birth. The very heterogeneous set of studies suggests detrimental effects of iron deficiency, and possibly also of overload, on other outcomes including child neurodevelopment. Studies are needed to determine clinically meaningful definitions of iron deficiency and overload in pregnancy.

## 1. Introduction

Iron is essential in pregnancy for maternal health as well as fetal growth and development [1,2]. Because iron deficiency anemia affects billions of people worldwide, many studies have focused on the impact of low hemoglobin levels on pregnancy outcome [2,3]. However, iron is not only required for hemoglobin synthesis, but is crucial for many additional processes including intracellular oxygen transport, cellular respiration, myelination, neurotransmitter production, and cell proliferation [4]. These processes may be compromised in iron deficiency, even in the absence of anemia [5]. Presently, iron status is monitored by biomarkers, such as serum ferritin, transferrin saturation, or soluble transferrin receptor concentration (sTfR) [6]. Clinical meaningful cut-offs for iron deficiency or iron overload in pregnancy and preferred methods have not yet been defined [7].

Iron deficiency in pregnancy generates risks for both the mother and her offspring. However, evidence for improved clinical outcomes upon routine iron supplementation other than for hematological parameters, is still lacking [2]. At the other end of the spectrum, human and animal studies have shown that excess iron is potentially harmful [7]. Iron excess causes oxidative stress, induces cellular damage, and is associated with a variety of health problems, including cardiovascular risk, pregnancy diabetes, and fetal complications [8,9,10]. Whilst the body is capable of limiting absorption of iron, controlled excretion of excess iron is not possible [10]. Therefore, the World Health Organization Guidelines recommending routine administration of iron supplements in pregnancy, without first clinically monitoring iron biomarkers, have been questioned [11]. Concern has been raised, especially in high income countries, over iatrogenic iron overload [9,12]. Two cohort studies have confirmed a U-shaped risk curve of maternal hemoglobin, reflecting a higher risk of adverse pregnancy outcomes among women with highest or lowest hemoglobin concentrations in pregnancy [13,14]. To what extend this non-linear adverse health effect is explained by the underlying iron status needs further study, as anemia may also occur in normal iron status, and high hemoglobin in pregnancy may reflect impaired plasma volume expansion [15]. However, the findings in early pregnancy, when hemodilution is still limited, suggest that other mechanisms, such as iron overload, may play a role.

Environmental factors in utero can alter fetal growth and organ development with a potential impact on the offspring’s longer-term health [16]. Studies on maternal iron deficiency and neurodevelopmental outcome suggest that iron status in pregnancy is such a key factor [17]. However, most studies have only examined the effect of iron supplementation and on short-term outcomes only [18]. A large systematic review on iron supplementation found an effect on maternal hemoglobin but not on child outcomes at birth [2]. Another review found a poor correlation between maternal and neonatal hemoglobin just after birth [19]. An overview of the longer-term consequences of maternal iron status on child health is still lacking. This is relevant to the discussion on general administration of iron supply versus personalized iron supplementation in pregnant women. The aim of this study was to systematically review and analyze the literature on maternal iron status in pregnancy and health outcomes in the offspring after birth.

## 2. Methods

### 2.1. Search Strategy and Study Selection

A systematic review was conducted with adherence to the Preferred Reporting Items of Systematic Review and Meta-Analysis (PRISMA) guidelines [20] and the pre-specified protocol was registered at PROSPERO (CRD42020162202). A search strategy was developed in collaboration with a clinical librarian to search Embase, Medline Ovid, Web of Science, Cochrane Central, and Google Scholar (Supplementary methods) from inception of the respective databases until 21 January 2021. After removing duplicates, two reviewers (H.G.Q.P. and F.C.) independently screened all titles and abstracts for eligibility. Full texts and reference lists were then screened independently. The final study selection was based on consensus between both reviewers and, if necessary, disagreement was resolved by arbitration by a third reviewer (M.J.V.).

Observational and intervention studies were eligible for inclusion if they investigated the association between maternal iron status at any stage of pregnancy, in relation to child health outcomes. Inclusion criteria were as follows: (1) iron status measured during pregnancy by (ratios of) serum ferritin, transferrin, transferrin saturation, or total iron binding capacity, iron, sTfR, or genetic proxies for any of these parameters; and (2) any child outcome being assessed after birth (at any age and into adulthood) related to health, development, or wellbeing. In the selection procedure, the following exclusion criteria were applied: (1) intervention studies in which assessment of iron status was done only before (not during/after) iron supplementation, as iron supplementation does not always lead to higher iron status depending on physiological and environmental factors; (2) availability of solely maternal hematological markers (e.g., hemoglobin, hematocrit, and/or erythrocytes), while more specific markers for iron status in pregnancy are lacking; (3) only fetal or birth outcomes (such as birth weight, preterm birth) or measurements in umbilical cord blood, while outcomes after birth are missing; (4) report not written in English; (5) animal studies; (6) abstracts, reviews, or commentaries not reporting original data; (7) studies whereby no abstract or full text was available (after internet search, national and international library requests, and addressing corresponding authors by email).

### 2.2. Data Extraction and Reporting

Standardized worksheets (Microsoft Excel™, Microsoft Corporation, Redmond, WA, USA, 2010) were used to systematically manage study selection, methodological quality assessment, and data extraction. The study characteristics included: year of publication, country income (low, lower-middle, upper-middle, high) [21], sample size, study design, use of iron supplementation, risk of bias, stage of pregnancy at iron measurement (first, second, and third trimester or peri-partum), maternal iron biomarker, cut-off used, outcome measure, and child age at outcome measurement. Mean ferritin values for the total study population or of the lower and higher category being used in the comparisons were recorded. If no mean was reported, we calculated the mean based on the reported median and measures of variability [22]. The effect of lower or higher iron status in the mother was categorized into harmful, neutral, or beneficial for the child, based on the reported results. Data and associations were extracted by one reviewer and then independently verified for accuracy and completeness by a second reviewer. If necessary, discrepancies were resolved by consensus with a third reviewer (M.J.V.)

### 2.3. Quality Assessment and Data Analysis

The methodological quality of each study was independently assessed by two reviewers H.G.Q.P. and F.C. using the quality score presented in the Newcastle Ottawa Scale [23]. In accordance with the guidelines, customized worksheets were designed for the cohort studies, intervention trials, and case-controlled studies. Studies were scored one to nine points and were categorized as low (7–9 points), medium (4–6 points), or high (1–3 points) risk of bias, based on appraisal of three domains, namely selection, comparability, and outcome. The following specific definitions were used: representativeness was considered high if the sample was representative of all pregnant women in the general community. Ascertainment of iron status was considered secure if adequate iron measures were assessed in blood and considered sufficient if objective indirect measures of iron status were used such as genetic proxies for iron markers. Stage of pregnancy was considered an important factor to control for in all studies [24], as was inflammation as ferritin is an acute phase protein that rises during inflammation [25].

Meta-analysis was considered only if 3 or more studies reported sufficient data on the association of the same iron marker in pregnancy and the same child health outcome. If an outcome was measured at multiple points in time, the measurement closest to the age of 6 months was used, as outcomes were most commonly reported at this age. Mean differences (MD) and associated 95% confidence intervals (CIs) in outcomes were calculated between children of mothers with iron deficiency (or low iron status) and children of mothers with higher iron status (normal or high range). Heterogeneity was evaluated by chi-square Q and I^2^ statistics. Given the high variance expected due to study heterogeneity, a random-effects model was used. The meta-analysis was done using Cochrane Review Manager software (REVMAN version 5.3, The Nordic Cochrane Centre, Copenhagen, Denmark) and R Software version 3.5.3. To explore the nature of heterogeneity, meta-regression was performed on the study characteristics. Multi-collinearity of study characteristics was evaluated in plots as well as correlation tests. The robustness of models was tested using permutation tests [26]. Publication bias was evaluated by funnel plots and Egger’s tests (cut-off *p*-value < 0.05).

## 3. Results

**Figure 1:**
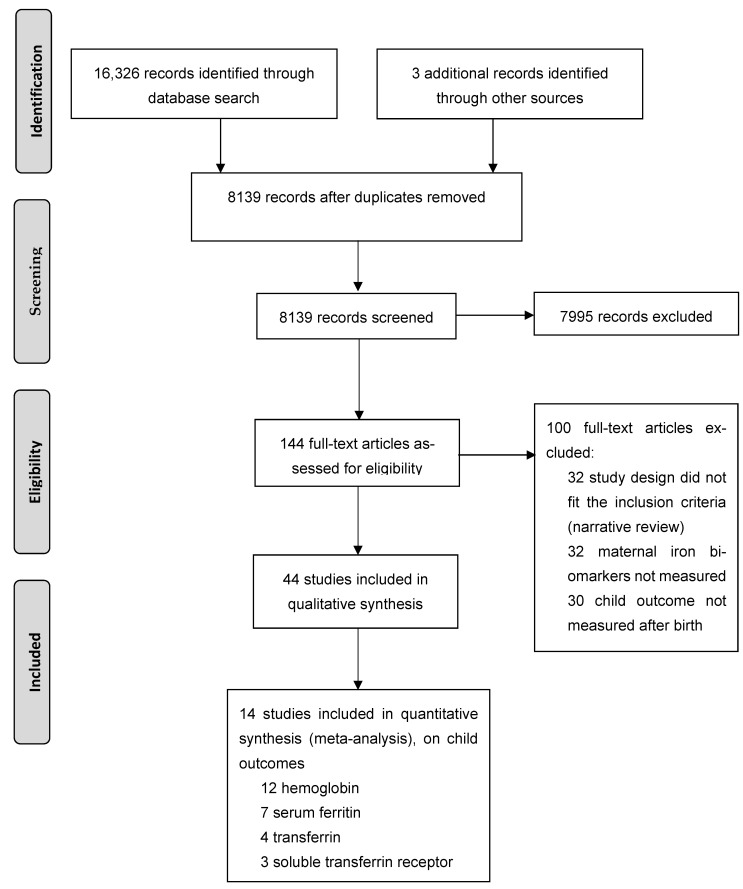
PRISMA flow diagram.

For the initial screening of titles and abstracts, 8139 studies, published between 1975 and January 2021, were identified. Of these, 142 papers were fully read and led to the identification of 41 studies meeting the inclusion criteria. Scanning reference lists led to the inclusion of three more studies, resulting in a total of 44 studies [27,28,29,30,31,32,33,34,35,36,37,38,39,40,41,42,43,44,45,46,47,48,49,50,51,52,53,54,55,56,57,58,59,60,61,62,63,64,65,66,67,68,69,70] (Figure 1).

Study characteristics are summarized in Table 1, Table 2, Table 3 and Table 4 and Supplementary Table S1. The reports describe sample sizes ranging from 26 to 94,209 per study, resulting in a total of 127,849 mother–child pairs included in this review. The studies originate from 23 countries and describe populations in low (9%), middle (43%), and high-income (48%) countries. Thirty-six papers (80%) describe cohort studies (31 prospective and four retrospective), five studies are randomized controlled trials with measurement of maternal iron status (during or after supplementation), and four are case control studies. Of note, in all clinical trials and in some of the cohort studies, iron supplementation occurred (*n* = 29; 66%). Iron supplementation was most commonly prescribed routinely from study inclusion until the end of pregnancy as 60 mg daily enteral doses, alone or in combination with folic acid (*n* = 7 studies).

### 3.1. Maternal Iron Status

Serum ferritin was used in 39 studies (89%) to classify maternal iron status (Table 1, Table 2, Table 3 and Table 4). Other biomarkers included serum iron, sTfR, or the sTfR:ferritin ratio. The lowest and the highest mean maternal serum ferritin were found in cohorts from Philippines (13.4 µg/L) [40] and India (62.6 µg/L) [41]. In many studies, mean ferritin concentrations were not specified (Table 1, Table 2, Table 3 and Table 4). Twenty-seven studies (61%) categorized women in order to compare iron deficiency or “low” to normal or “higher” iron status, whilst others categorized according to supplementation or health status. As no definitions are universally agreed upon, we found different cut-offs being applied to ferritin ranging from ferritin < 9 µg/L in an American study [27] to <50 µg/L in a Finnish study [34]. Mostly, the lower category was based on a ferritin <12 (*n* = 10 studies). Studies not comparing categories analyzed biomarkers as continuous variables in linear models. The majority of studies (91%) focused on low(er) maternal iron status or iron deficiency, whilst iron status above normal or iron excess was the focus in four studies only [31,67,68,69]. Four recent studies used maternal common genetic variants as proxies for iron stats [46,62,65,69]. These included twelve single nucleotide polymorphisms (SNPs) at different locations associated with lower iron status and two in the hemochromatosis (*HFE*) gene associated with iron overload. This gene is involved in intestinal absorption and cellular iron uptake and may impact trans-placental transport of iron. [71].

Maternal measurements were timed throughout pregnancy, with most of them occurring during the third trimester or at birth. Of the 10 studies that examined the first trimester of pregnancy, reflecting the period of organogenesis, only four explored outcomes beyond infancy [53,58,60,64]. One found impaired memory in 5-year-old children of mothers with anorexia nervosa and low ferritin levels [53]. Another showed associations between maternal lower iron status, child wheezing, and impaired lung function [64]. The third found that lower maternal ferritin levels were associated with lower child scores in memory and executive functioning at 7 years of age [58]. The last one found an association of lower maternal ferritin and symptoms of poor attention in 4- to 5-year-old boys, though not in girls [60].

### 3.2. Quality Assessment

Risk of bias scores ranged from 3 to 9 (median 8), with most studies having a low (*n* = 32; 71%) or medium (*n* = 10; 22%) risk of bias (Table 1). Study samples appeared reasonably representative of pregnant women in the general population, however, nine studies had no description of the derivation of the cohort. Blind assessment of the outcome was described in 12 cohort studies. The most common risk of bias was the lack of control for inflammation whilst studying ferritin, an issue that was properly addressed in only 14 studies. These studies either adjusted the cut-off of ferritin in case of inflammation (*n* = 2) [40,49], excluded women with high inflammation markers (*n* = 4) [38,41,57,58], excluded cases with high ferritin (*n* = 1) [51], statistically corrected for inflammation (*n* = 1) [33], used genetic instrumental variables (*n* = 4) [46,62,65,69], or used a combination of these methods (*n* = 2) [47,55]. From eight studies, data on sTfR were available, which is a marker that inversely correlates with the amount of iron available for erythropoiesis and is less affected by inflammation [3].

### 3.3. Child Outcome

Details on study designs (year of publication, country, country income, and risk of bias), maternal iron status (biomarker used, ferritin cut-offs, or mean ferritin levels, and pregnancy stage), child outcomes (description and age), and associations observed (direction of effect) are summarized in Table 1, Table 2, Table 3 and Table 4. These also show whether the association of a higher maternal iron status, as compared to iron deficiency or relative to lower iron state in linear models, correlates with a beneficial, detrimental, or indifferent outcome in the child.

The reported child health outcomes varied widely. Outcome was predominantly measured in early infancy (*n* = 32; 73%), but ages ranged from 24 h after birth until school-age. Only one study focused on adult outcomes, reporting no association between maternal iron status and off-spring blood pressure and adiposity at 40 years of age [62]. Sixteen studies examined the iron status or hemoglobin concentrations (or both) of children, though not after 12 months of age (Table 1, Table 2, Table 3 and Table 4). Twenty-one studies reported more clinical or functional outcomes, including different aspects of neurodevelopment (*n* = 18) and hearing status (*n* = 3) [56,57,58] (Table 3). The remaining ten studies examined cardiovascular risk factors (*n* = 2) [62,63], pulmonary issues (*n* = 2) [64,65], and all others examined bone mass, retinopathy of prematurity, infant mortality, neonatal sepsis, infant growth, and Type 1 diabetes, respectively (Table 4).

**Figure 2:**
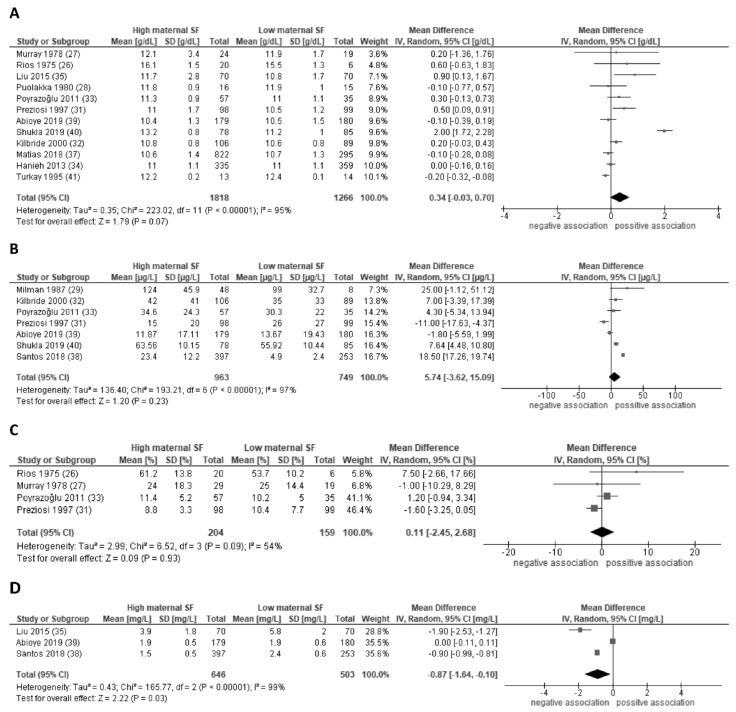
Forest plot of the associations of maternal serum ferritin levels during pregnancy with child hemoglobin concentrations (**A**), child serum ferritin concentrations (**B**), child transferrin saturation (**C**), and child soluble transferrin receptor concentrations (**D**). Random-effect meta-analysis showing the pooled mean difference child hemoglobin concentrations comparing high and low maternal serum ferritin in pregnancy. CI, confidence interval; IV, inverse variation; SF, serum ferritin; DF, degrees of freedom.

The 16 studies on child iron status or anemia were mostly 83% carried out in high and middle-income countries. Sample sizes of these studies ranged from 26 to 1194 mother-child pairs. The majority had a moderate or low risk of bias. Fourteen studies provided sufficient data for meta-analysis on the outcomes of four iron-related hematological parameters. No significant association of maternal iron status (i.e., serum ferritin) and child hemoglobin, ferritin, or transferrin saturation was found (Table 2, Figure 2A–C). The only consistent association between maternal ferritin and child iron status was found in sTfR (Figure 2D). Heterogeneity was generally high, with I^2^ ranging from 54% for the studies on child transferrin saturation to over 95% for the other outcomes. Meta-regression suggests that associations with child hemoglobin are stronger in more recent studies, whereby mothers were tested later in pregnancy and the child outcomes were assessed at a younger age (combined explained variance 96%, *p* < 0.01). Heterogeneity in the child ferritin studies was mainly explained by stronger associations found in higher income countries (explained variance 77%, *p* = 0.02). Study design, iron supplementation, and quality did not significantly explain variance in effect estimates (Supplementary Table S2). Sensitivity analyses in subgroups to assess the robustness of our findings did not materially change our conclusions. Publication bias was considered unlikely (Supplementary Figure S1).

Neurodevelopmental outcomes were the focus of 20 studies (45%) with sample sizes ranging from 63 to 11,696 (Table 3). All of these studies were conducted in high- and middle-income countries, except for one study from Benin [49]. Only one of the studies on neurodevelopment, published in 1986, showed a high risk of bias [50]. Except for one Mendelian randomization study [46], all neurodevelopmental studies measured serum ferritin in pregnancy. The neurodevelopmental outcomes examined included cognition, motor development, and child behavior, evaluated in infancy and/or childhood up to 8 years of age [46]. The majority of studies used standardized neurodevelopmental tests, including cognitive, motor, verbal, memory functioning, and neonatal behavior. Results varied, with only 12 out of the 20 studies reporting better neurodevelopmental outcome in children from mothers with higher iron status as compared to mothers whose iron status was considered low. Two of the three studies on auditory brainstem response, measured between 24 h and 3 months of age, found a positive association of maternal ferritin concentrations in pregnancy and child’s hearing later in later life [56,57]. Studies on the various other outcomes are summarized in Table 4.

We identified four reports on three studies reporting on high maternal iron status, as opposed to normal, and its possible relationship with adverse outcomes in children [31,67,68,69]. In a Vietnamese cluster randomized trial, ferritin was higher in women with daily dosage, as compared to those with intermittent dosage of iron supplementation [31,67]. Follow-up at 6 months of age showed lower cognitive scores and lower length for age in children of mothers with higher ferritin levels in pregnancy [31,67]. The effect on infant growth was largely mediated through birth weight, which was lower in mothers with higher ferritin [67]. The authors explain their findings by deleterious fetal effects of iron supplementation in women who already have normal iron stores. Harmful effects of iron supplementation in women without iron deficiency was also suggested in a very large Norwegian cohort [69]. They found an increased risk of childhood diabetes Type I in children of mothers with iron supplementation or with common genetic variants in the *HFE* gene that are linked to high/intermediate iron stores. Their findings likely reflect intra-uterine effects, as the association was not explained by the HLE genotype of the child [69]. The fourth study on high maternal iron status reported an association between elevated ferritin levels and neonatal sepsis among women with premature rupture of membranes, likely to be explained by an acute phase response during infection [68]. Studies specifically exploring a U-shaped curve for risk were not found. None of the included studies applied non-linear models to identify risks of both iron deficiency and iron excess on the same outcome. The only study comparing three maternal ferritin categories was in African women under famine conditions back in 1978, who were without supplementation. No U-shaped curve for child hematological measures was found [28].

## 4. Discussion

In this systematic review on maternal iron status in pregnancy, we summarize 44 studies on a wide range of child health outcomes measured shortly after birth into adulthood. The main finding of the review is that a normal or higher maternal iron status in pregnancy, as compared to iron deficiency or lower iron status, was either associated with child health benefits (46% of studies) or showed no significant association at all (48%). Meta-analyses of studies on biochemical outcomes found a significant negative association between maternal iron status and child sTfR levels but no association with child ferritin, transferrin saturation, and hemoglobin. As sTfR is a reliable biomarker of low iron status which is less affected by inflammation, our findings suggest that maternal and child iron status are indeed related, which is in line with earlier reviews on short-term effects including cord blood studies [72,73,74,75]. After infancy, studies mainly focused on neurodevelopmental outcomes, with 12 out of 20 studies reporting better outcomes in children of mothers with higher iron status and one reporting harmful effects of higher iron status [31]. These outcomes included cognition, motor function, language, and memory. Additionally, child hearing may benefit from a higher maternal iron status. The magnitude of the global effects of iron deficiency remains underexposed, with only one of the neurodevelopmental studies being carried out in a low-income country.

The finding that in nearly half of the studies, maternal iron status is not associated with child health outcome, may have different explanations.

(1) It is possible that maternal iron status is not associated with these outcomes because the studied outcomes vary in their sensitivity to this exposure. It could be that compensating regulatory mechanisms may in fact protect the child from disturbance of the maternal iron balance. For example, the placenta can actively transport iron from the mother to the fetus, thereby protecting the latter from iron deficiency, even at the expense of maternal iron stores [24].
(2) The resulting effects may be too modest to be detected at a later age, especially in small studies. Indeed, our meta-regression on hemoglobin studies suggests weaker associations when maternal and child hemoglobin measurements are further apart. If effects are modest at a later age, the clinical impact is likely to be considered low.
(3) Existing effects may have been missed due to the high heterogeneity regarding study populations, laboratory methods, and cut-offs applied. Definitions of iron deficiency varied across studies and within countries. This is inevitable as worldwide applicable definitions of iron deficiency or iron overload in pregnancy have yet to be defined.
(4) Timing of measurements during pregnancy is important as iron stores change dramatically during the course of pregnancy. Trimester-specific cut-offs that consider the physiological increases in blood volume, hemodilution, and iron requirements of the placenta and fetus are needed. Results may have differed if measurements were more consistent across studies [76].
(5) During pregnancy, iron supplementation, as well as individual differences in body weight, dietary intake, micronutrient deficiencies, and iron absorption, may mask effects of iron deficiency or otherwise in both mother and child [77]. Additionally, iron intake during earlier infancy including breastfeeding practices and iron enrichment or supplementation might also influence our results and might explain differences between countries [15]. These potential confounders or mediators were generally not analyzed, except for eleven studies that took breastfeeding into account.
(6) Another very likely explanation is that results are biased by inflammation, which is largely not reported [78]. This is of specific relevance in regions with high prevalence of (chronic) infections, like tuberculosis, malaria, and other parasitic infections [6]. During states of inflammation, the acute phase protein ferritin rises markedly, even in the case of iron deficiency. That may explain why no effect was detected on child iron biomarkers except for sTfR, which is controlled by iron availability and not by inflammation [3].
(7) Another possibility is that maternal iron effects are non-linear, as hypothesized earlier based on the U-shaped risk curve of maternal hemoglobin [13]. If iron deficiency and iron overload are both harmful, effects may be missed in linear statistical models or in studies comparing two categories that include both these extremes. We conclude that there is little evidence to support or reject a U-shaped risk curve of maternal iron status on specific outcomes.

With only four reports being found on high or excessive maternal iron and all of them reporting adverse outcomes in the offspring [31,67,68,69], we propose that more research on long-term effects of iron overload is urgently needed, especially in populations with low prevalence of iron deficiency. The possible harmful effects of high maternal iron status on the fetus supports region-specific individualization of iron supplementation to prevent iatrogenic iron overload. The findings call for a global approach in establishing clinically meaningful definitions of iron deficiency and overload that are pregnancy-stage–specific, and are based on short- and long-term outcomes.

### Strengths and Limitations

To our knowledge, this study is unique in providing a comprehensive systematic review and meta-analysis of maternal iron status during pregnancy and child health outcomes after birth. Strengths of our analysis are the broad search strategy in accordance with current guidelines, including both observational and interventional studies. Although, this study has some limitations. By excluding studies not written in English, a language bias may have been introduced. By applying no restriction on publication date or on laboratory techniques used, we may also have selected lower-quality studies that diluted the effects. We decided to exclude studies on maternal hemoglobin that did not report on specific iron biomarkers. Hemoglobin is often used as a proxy for iron status, but it is neither a sensitive nor a specific measure of iron deficiency [79]. That is because hemoglobin is not solely determined by iron status, but also by residential altitude [80], ethnicity, nutritional status, hemoglobinopathies, as well as infectious and chronic diseases [81]. Because iron has many other biochemical functions in all cell types, we expected that child health effects of the maternal iron status are of critical importance beyond the effects on hemoglobin. We cannot rule out that by focusing on maternal iron status and not on maternal anemia, we may have missed important studies that used hemoglobin as a proxy for iron status. However, a very recent review on maternal hemoglobin in pregnancy did not find data on long-term child health outcomes [82].

Furthermore, factors explaining the differences in maternal iron status did not fall within the scope of this review. In two-thirds of studies, iron supplementation was involved, but other unmeasured factors, including socioeconomic status, health status, diet, and genetic factors may also have played a role. The risk of confounding needs to be considered when interpreting the results of the predominantly observational studies. Additional limitations include high heterogeneity amongst the studies and the absence of assessment of the likely causal pathways, limiting the ability to form solid conclusions. Based on the current literature, it is not feasible to define the physiologic range or to determine useful clinical cut-offs for adverse iron status in pregnancy.

## 5. Conclusions

We found that maternal iron status in pregnancy is likely to be associated with child iron status after birth, but only to a modest extent, with not all iron biomarkers following this trend. Findings from a very heterogeneous set of studies may suggest beneficial effects of higher maternal iron status on other outcomes including child neurodevelopment. Long-term effects of maternal iron status above normal need further exploration. Our analysis highlights the need for more high-quality studies that look beyond hemoglobin concentrations and beyond birth, thereby determining the physiological range and defining outcome-based definitions of iron deficiency and iron overload in pregnancy.

## Figures and Tables

**Table 1:** Studies on maternal iron status and child iron status after birth.

Scheme	Year	Country	Country Income	Design (S)	Risk of Bias	*n*	Population	Back-Ground SF, µg/L	Statistics	Mother			Child	
										Iron Biomarkers	Categories Based on	Stage	Outcome	Age
No association														
Rios [27] *	1975	USA	H	Cohort (S)	M	26	General	NR	*T*-test	SF, Fe, TSAT, TIBC	SF < 9 µg/L	PP	SF, TSAT	6 d
Murray [28] *	1978	Niger	L	Cohort	M	49	Famine conditions	NA	NR	Fe, TSAT	TSAT < 10% vs. TSAT > 60%	PP	SF, TSAT	6 m
Kilbride [29] *	2000	Jordan	U-M	Case control (S)	H	195	Refugees	15.2 ‡	*T*-test	SF, Fe, TSAT, (TIBC)	Anemia, SF < 12 µg/L	PP	SF	3, 6, 9, 12 m
Poyrazoğlu [30] *	2011	Turkey	U-M	Cohort (S)	M	92	General	33 ‡	ANOVA	SF, Fe, TSAT, TIBC	Anemia and (S)	II, PP	SF, TSAT	3, 6, 12 m
Hanieh [31] *	2013	Vietnam	L-M	Clinical trial (S)	L	1168	General	28.4 §	MLR	SF, (sTfR, sTfR/SF)	(S), SF < 15 µg/L	III	SF	6 m
Kulik [32] *	2016	Poland	H	Cohort (S)	M	44	General	22 ‡	corr	SF, sTfR	SF < 15 µg/L	PP	SF	3 d
Matias [33] *	2018	Bangladesh	L-M	Clinical trial (S)	L	1117	General	NR	MLR	SF, sTfR	SF < 12 µg/L	III	SF	6 m
Positive association														
Puolakka [34] *	1980	Finland	H	Cohort (S)	H	47	General	NR	MWU	SF (Fe, TF, TSAT, TIBC)	SF < 50 µg/L	PP	SF	6 m
Milman [35] *	1987	Denmark	H	Cohort (S)	M	56	General	21 †	MWU	SF (Fe, TF, TSAT)	SF < 15 µg/L	PP	SF	5 d
Morton [36]	1988	UK	H	Cohort (S)	M	51	General	NR	*T*-test	SF	SF < 10 µg/L	III	SF	6 m
Preziosi [37] *	1997	Niger	L	Clinical trial (S)	M	197	General	NR	*T*-test	SF (Fe, TSAT)	SF < 12 µg/L	II, PP	SF (Fe, TSAT)	3, 6 m
Liu [38] *	2015	China	U-M	Cohort	L	140	General	19.9 ‡	*T*-test	SF, sTfR	Anemia	III	sTfR (SF)	6 m (42 d, 4 m)
Santos [39] *	2018	China	U-M	Clinical trial (S)	L	1194	General	NR	MLR	SF, sTfR		II, III	SF, sTfR	9 m
Abioye [40] *	2019	Philippines	L-M	Nested-cohort (S)	L	359	Women with schistosomiasis and anemia	13.4 ‡	MLR	SF, (sTfR)	SF < 12 µg/L	III	SF, (sTfR)	6 m (12 m)
Shukla [41] *	2019	India	L-M	Cohort	L	163	General	62.6 ‡	T-test, corr	SF	Anemia	PP	SF	14 wk

Country income according to World Bank classification (https://datahelpdesk.worldbank.org/knowledgebase/articles/906519 accessed on 20 July 2020 ); corr, correlation; d, Days; I, II, III, gestational trimester; H, high; L, low; L-M, lower-middle; U-M, upper-middle; MWU, Mann–Whitney U-test; M, medium; m, months; MLR, multiple linear regression; NA, not applicable; PP, peri-partum; SF, serum ferritin represents reported (or calculated); sTfR, soluble transferrin receptor; (S), maternal iron supplementation, TF, transferrin; TSAT, transferrin saturation; TIBC, total iron binding capacity; wk, week; y, year. * Studies reporting multiple outcomes; †, median; ‡, mean; §, geometric mean; values in the total study population. Studies are grouped by the direction of the association between maternal iron status and child hemoglobin.

**Table 2:** Studies on maternal iron status and child hemoglobin concentrations after birth.

Study	Year	Country	Country Income	Design (S)	Risk of Bias	*n*	Population	Back-ground SF, µg/L	Statistics	Mother			Child	
										Iron Biomarkers	Categories Based on	Stage	Outcome	Age
No association														
Rios [27] *	1975	USA	H	Cohort (S)	M	26	General	NR	*T*-test	SF, Fe, TSAT, TIBC	SF < 9 µg/L	PP	Hemoglobin	6 d
Murray [28] *	1978	Niger	L	Cohort	M	49	Famine conditions	NA	NR	Fe, TSAT	TSAT < 10% vs. TSAT > 60%	PP	Hemoglobin	6 m
Puolakka [34] *	1980	Finland	H	Cohort (S)	H	47	General	NR	MWU	SF (Fe, TF, TSAT, TIBC)	SF < 50 µg/L	PP	Hemoglobin	6 m
Milman [35] *	1987	Denmark	H	Cohort (S)	M	56	General	21 †	MWU	SF (Fe, TF, TSAT)	SF < 15 µg/L	PP	Hemoglobin	5 d
Turkay [42]	1995	Turkey	U-M	Cohort	M	27	General	13.5 ‡	*T*-test, corr	SF, Fe, TSAT	SF < 12 µg/L	II, III	Hemoglobin	3 m
Preziosi [37] *	1997	Niger	L	Clinical trial (S)	M	197	General	NR	*T*-test	SF (Fe, TSAT)	SF < 12 µg/L	II, PP	Hemoglobin	3, 6 m
Poyrazoğlu [30] *	2011	Turkey	U-M	Cohort (S)	M	92	General	33 ‡	ANOVA	SF, Fe, TSAT, TIBC	Anemia and (S)	II, PP	Hemoglobin	3, 6, 12 m
Hanieh [31] *	2013	Vietnam	L-M	Clinical trial (S)	L	1168	General	28.4 §	MLR	SF (sTfR sTfR/SF)	(S), SF < 15 µg/L	III	Hemoglobin	6 m
Liu [38] *	2015	China	U-M	Cohort	L	140	General	19.9 ‡	*T*-test	SF, sTfR	Anemia	III	Hemoglobin	42 d, 4 m, 6 m
Kulik [32] *	2016	Poland	H	Cohort (S)	M	44	General	22 ‡	corr	SF, sTfR	SF <15 µg/L	PP	Hemoglobin	3 d
Matias [33] *	2018	Bangladesh	L-M	Clinical trial (S)	L	1117	General	NR	MLR	SF, sTfR	SF <12 µg/L	III	Hemoglobin	6 m
Positive association														
Kilbride [29] *	2000	Jordan	U-M	Case control (S)	H	195	Refugees	15.2 ‡	*T*-test	SF, Fe, TSAT (TIBC)	Anemia, SF <12 µg/L	PP	Hemoglobin	9, 12 m (3, 6m)
Santos [39] *	2018	China	U-M	Clinical trial (S)	L	1194	General	NR	MLR	SF, sTfR		II, III	Hemoglobin	9 m
Abioye [40] *	2019	Philippines	L-M	Nested-Cohort (S)	L	359	Women with schistosomiasis and anemia	13.4 ‡	MLR	SF, (sTfR)	SF <12 µg/L	III	Hemoglobin	6 m (12 m)
Shukla [41] *	2019	India	L-M	Cohort	L	163	General	62.6 ‡	*T*-test, corr	SF	Anemia	PP	Hemoglobin	14 wk

Country income according to World Bank classification (https://datahelpdesk.worldbank.org/knowledgebase/articles/906519 accessed on 20 July 2020 ); corr, correlation; d, Days; I, II, III, gestational trimester; H, high; L, low; L-M, lower-middle; U-M, upper-middle; MWU, Mann–Whitney U-test; M, medium; m, months; MLR, multiple linear regression; NA, not applicable; PP, peri-partum; SF, ferritin represents reported (or calculated); sTfR, soluble transferrin receptor; (S), maternal iron supplementation; TF, transferrin; TSAT, transferrin saturation; TIBC, total iron binding capacity; wk, week; y, year. * Studies reporting multiple outcomes; †, median; ‡, mean; §, geometric mean; values in the total study population. Studies are grouped by the direction of the association between maternal iron status and child hemoglobin.

**Table 3:** Studies on maternal iron status and child neurobehavioral outcomes.

Study	Year	Country	Country Income	Design (S)	Risk of Bias	*n*	Population	Background SF, µg/L	Statistics	Mother			Child	
										Iron Biomarkers	Categories Based on	Stage	Outcome	Age
No association														
Zhou [43]	2006	Australia	H	Clinical trial (S)	L	430	General	14 ‡	*T*-test	SF	(S), SF < 12 µg/L	III	Intelligence: reasoning, memory	4 y
Davidson [44]	2008	Seychelles	H	Cohort (S)	L	229	General	NR	MLR	SF, sTfR		II	Psychomotor	5, 9, 25, 30 m
Rioux [45]	2011	Canada	H	Cohort (S)	M	63	General	NR	MLR	SF	SF < 10 µg/L	III	Cognition	6 m
Lewis [46]	2014	UK	H	Cohort (S)	L	11,696	General	NA	MLR	IV			Cognition	8 y
Tran [47]	2014	Vietnam	L-M	Cohort	L	418	General	17.3 ‡	MLR	SF	SF < 15 µg/L	I, III	Motor	6 m
Lou [48]	2015	China	U-M	Cohort	L	69	General	17.4 ‡	ANCOVA	SF	SF < 16 µg/L	III	Auditory brainstem response	3, 10 m
Mireku [49]	2016	Benin	L	Cohort (S)	L	636	General	NR	MLR	SF		II, PP	Cognition, motor, language, visual, perception	12 m
Higher maternal iron status associated with adverse child outcome														
Hanieh [31] *	2013	Vietnam	L-M	Clinical trial (S)	L	713	General	28.4 §	MLR	SF, (sTfR, sTfR/SF)	(S), SF < 15 µg/L	III	Cognition, language, motor	6 m
Higher maternal iron status associated with better child outcome														
Vaughn [50]	1986	USA	H	Cohort (S)	H	115	General	NR	*T*-test	TIBC (SF, Fe)	Irritability	III	(Neonatal behavior), Irritability	3d (5 m)
Hernández [51]	2011	Spain	H	Cohort (S)	L	216	General	NR	MLR	SF, TSAT	SF < 12 µg/L	I, II, III	Neonatal behavior	Neonatal period
Tran [52]	2013	Vietnam	L-M	Cohort	L	378	General	NR	MLR	SF	SF < 15 µg/L	I, III	Cognition	6 m
Koubaa [53]	2015	Sweden	H	Cohort	L	116	Women with eating disorders	42.7 ‡	corr	SF	SF < 20 µg/L	I	Memory	5 y
Park [54]	2016	Philippines	L-M	Nested-cohort	L	370	Women with schistosomiasis	6.2 §	MLR	SF, sTfR/SF (sTfR)		III	Cognition, language, motor	12 m
Berglund [55]	2017	Spain	H	Cohort (S)	L	331	General	16.7 ‡	ANCOVA	SF, TSAT	SF < 15 µg/L	III, PP	Cognition, language, motor	18 m
Santos [39] *	2018	China	U-M	Clinical trial (S)	L	1194	General	NR	MLR	SF, sTfR		II, III	Motor	9 m
Choudhury [56]	2015	India	L-M	Cohort	L	90	General	36.9 ‡	MLR	SF		PP	Auditory brainstem response	24 to 48 h
ElAlfy [57]	2018	Egypt	L-M	Case control	L	100	General	55.6 ‡	*T*-test	SF (Fe, TSAT, TIBC)	SF < 15 µg/L	PP	Auditory brainstem response	48 h, 3 m
Arija [58]	2019	Spain	H	Cohort (S)	L	2032	General	NR	MLR	SF	SF < 12 µg/L	I	Memory, attention, flexibility and inhibition	7 y
Kupsco [59]	2020	Mexico	U-M	Cohort (S)	L	571	General	36.7 ‡	GAM	SF	SF < 15 µg/L	II, III, PP	Memory, motor, cognition	4–6 y
Santa-Marina [60]	2020	Spain	H	Cohort	L	1095	General	35.9 ‡	PRM	SF		I	Symptoms of Attention deficit/hyperactivity disorder	4–5 y

Country income according to World Bank classification (https://datahelpdesk.worldbank.org/knowledgebase/articles/906519 accessed on 20 July 2020 ); corr, correlation; d, Days; I, II, III, gestational trimester; GAM, generalized additive models; H, high; IV, instrumental variable; L, low; L-M, lower-middle; U-M, upper-middle; MWU, Mann–Whitney U-test; M, medium; m, months; MLR, multiple linear regression; NA, not applicable; PP, peri-partum; PRM, Poisson regression model; SF, ferritin represents reported (or calculated); sTfR, soluble transferrin receptor; (S), maternal iron supplementation, TF, transferrin; TSAT, transferrin saturation; TIBC, total iron binding capacity; wk, week; y, year. * Studies reporting multiple outcomes; ‡, mean; §, geometric mean; values in the total study population. Studies are grouped by the direction of the association between maternal iron status and child neurobehavioral outcomes.

**Table 4:** Studies on maternal iron status and other child outcomes after birth.

Study	Year	Country	Country Income	Design (S)	Risk of Bias	*n*	Population	Back-ground SF, µg/L	Statistics	Mother			Child	
										Iron Biomarkers	Categories Based on	Stage	Outcome	Age
Cardiovascular and bone outcomes														
Ganpule [61]	2006	India	L-M	Cohort (S)	L	797	General	NR	MLR	SF	SF < 12 µg/L	II	Bone mass	6 y
Alwan [62]	2012	UK	H	Cohort	L	348	General	NA	MLR	IV			Blood pressure, adiposity	40–41 y
Alwan [63]	2015	UK	H	Cohort (S)	L	362	General	37.5 ‡	MLR	SF, sTfR, sTfR/sF	SF < 15 µg/L	I	Arterial Stiffness	6 wk
Pulmonary outcomes														
Nwaru [64] *	2014	UK	H	Cohort (S)	L	157	General	28.4 ‡	MLR	SF, sTfR, sTfR/sF	SF < 15 µg/L	I, PP	Wheeze, allergy	1, 2, 5, 10 y
Nwaru [64] *	2014	UK	H	Cohort (S)	L	157	General	28.4 ‡	MLR	SF, sTfR, sTfR/sF	SF < 15 µg/L	I, PP	Lung function	1, 2, 5, 10 y
Bédard [65]	2018	UK	H	Cohort (S)	L	6002	General	NA	MLR	IV			Lung function	7.5 y
Miscellaneous outcomes														
Abioye [66]	2016	Tanzania	L	Cohort (S)	L	600	General	47.5 †	MLogR	SF, sTfR	SF ≤ 12 µg/L	I, II, PP	Infant mortality	6 w
Hanieh [67]	2015	Vietnam	L-M	Nested-cohort (S)	L	1046	General	28 †	MLR	SF		I, III	Infant growth	6 w, 6 m
Goldenberg [68]	1998	USA	H	Nested-cohort	L	223	Women with PRM	38.3 ‡	MLogR	SF		II, PP	Neonatal sepsis	Neonatal period
Størdal [69]	2018	Norway	H	Case control (S)	L	94,209	General	NA	CPHR	IV			Diabetes type 1	8 to 17 y
Dai [70]	2015	Turkey	U-M	Case control	M	254	General	24.3 ‡	T-test	SF, Fe	Stage of ROP	III	Retinopathy of prematurity	Infancy

Country income according to World Bank classification (https://datahelpdesk.worldbank.org/knowledgebase/articles/906519 accessed on 20 July 2020 ); corr, correlation; CPHR, Cox proportional hazard regression; d, days; I, II, III, gestational trimester; H, high; IV, instrumental variable; L, low; L-M, lower-middle; U-M, upper-middle; MWU, Mann–Whitney U-test; M, medium; m, months; MLR, multiple linear regression; MLogR, multiple logistic regression; NA, not applicable; PP, peri-partum; PRM, premature rupture of the membranes; ROP, retinopathy of prematurity; SF, ferritin represents reported (or calculated); sTfR, soluble transferrin receptor; (S), maternal iron supplementation; TF, transferrin; TSAT, transferrin saturation; TIBC, total iron binding capacity; wk, week; y, year. * Studies reporting multiple outcomes; †, median; ‡, mean; values in the total study population.

## Supplementary Materials

The following are available online at https://www.mdpi.com/article/10.3390/nu13072221/s1, Figure S1: Funnel plots for studies on child ferritin and haemoglobin, Table S1: Estimated risk of bias of included studies, Table S2: Meta-regression on studies on child haemoglobin or ferritin concentrations.

## Abbreviations

CI	Confidence Interval
IV	Instrumental Variable
MD	Mean Differences
PRISMA	Preferred Reporting Items of Systematic Review and Meta-Analysis Guidelines
SF	Serum Ferritin
sTfR	Soluble Transferrin Receptor Concentration
TIBC	Total Iron Binding Capacity
TSAT	Transferrin Saturation

## References

[1] Cerami C. (2017). Iron Nutriture of the Fetus, Neonate, Infant, and Child. Ann. Nutr. Metab..

[2] Peña-Rosas J.P., De-Regil L.M., Garcia-Casal M.N., Dowswell T. Daily oral iron supplementation during pregnancy. Cochrane Database Syst. Rev..

[3] WHO (2004). Assessing the Iron Status of Populations.

[4] Calder P.C. (2010). Iron and Health.

[5] Abbaspour N., Hurrell R., Kelishadi R. (2014). Review on iron and its importance for human health. J. Res. Med. Sci..

[6] Lynch S., Pfeiffer C.M., Georgieff M.K., Brittenham G., Fairweather-Tait S., Hurrell R.F., McArdle H.J., Raiten D.J. (2018). Biomarkers of Nutrition for Development (BOND)-Iron Review. J. Nutr..

[7] Daru J., Allotey J., Pena-Rosas J.P., Khan K.S. (2017). Serum ferritin thresholds for the diagnosis of iron deficiency in pregnancy: A systematic review. Transfus. Med..

[8] Gambling L., Dunford S., Wallace D.I., Zuur G., Solanky N., Srai S.K., McArdle (2003). Iron deficiency during pregnancy affects postnatal blood pressure in the rat. J. Physiol..

[9] Kataria Y., Wu Y., Horskjær P.d.H., Mandrup-Poulsen T., Ellervik C. (2018). Iron Status and Gestational Diabetes-A Meta-Analysis. Nutrients.

[10] Woodman A.G., Care A.S., Mansour Y., Cherak S.J., Panahi S., Gragasin F.S., Bourque S.L. (2017). Modest and Severe Maternal Iron Deficiency in Pregnancy are Associated with Fetal Anaemia and Organ-Specific Hypoxia in Rats. Sci. Rep..

[11] World Health Organization (2012). Guideline: Daily Iron and Folic Acid Supplementation in Pregnant Women.

[12] Brannon P.M., Taylor C.L. (2017). Iron Supplementation during Pregnancy and Infancy: Uncertainties and Implications for Research and Policy. Nutrients.

[13] Dewey K.G., Oaks B.M. (2017). U-shaped curve for risk associated with maternal hemoglobin, iron status, or iron supplementation. Am. J. Clin. Nutr..

[14] Gaillard R., Eilers P.H.C., Yassine S., Hofman A., Steegers E.A.P., Jaddoe V.W.V. (2014). Risk Factors and Consequences of Maternal Anaemia and Elevated Haemoglobin Levels during Pregnancy: A Population-Based Prospective Cohort Study. Paediatr. Perinat. Epidemiol..

[15] Georgieff M.K., Krebs N.F., Cusick S.E. (2019). The Benefits and Risks of Iron Supplementation in Pregnancy and Childhood. Annu. Rev. Nutr..

[16] Kwon E.J., Kim Y.J. (2017). What is fetal programming?: A lifetime health is under the control of in utero health. Obstet. Gynecol. Sci..

[17] Doom J.R., Georgieff M.K. (2014). Striking While the Iron is Hot: Understanding the Biological and Neurodevelopmental Effects of Iron Deficiency to Optimize Intervention in Early Childhood. Curr. Pediatrics Rep..

[18] Malinowski A.K., D’Souza R., Khan K.S., Shehata N., Malinowski M., Daru J. (2019). Reported Outcomes in Perinatal Iron Deficiency Anemia Trials: A Systematic Review. Gynecol. Obstet. Invest..

[19] Sanni O.B., Chambers T., Li J.H., Rowe S., Woodman A.G., Ospina M.B., Bourque S.L. (2020). A systematic review and meta-analysis of the correlation between maternal and neonatal iron status and haematologic indices. EClinicalMedicine.

[20] Liberati A., Altman D.G., Tetzlaff J., Mulrow C., Gotzsche P.C., Ioannidis J.P., Clarke M., Devereaux P.J., Kleijnen J., Moher D. (2009). The PRISMA statement for reporting systematic reviews and meta-analyses of studies that evaluate healthcare interventions: Explanation and elaboration. BMJ.

[21] Lotfaliany M., Akbarpour S., Zafari N., Mansournia M.A., Asgari S., Azizi F., Hadaegh F., Khalili D. (2018). World Bank Income Group, Health Expenditure or Cardiometabolic Risk Factors? A Further Explanation of the Wide Gap in Cardiometabolic Mortality Between Worldwide Countries: An Ecological Study. Int. J. Endocrinol. Metab..

[22] Wan X., Wang W., Liu J., Tong T. (2014). Estimating the sample mean and standard deviation from the sample size, median, range and/or interquartile range. BMC Med. Res. Methodol..

[23] Zeng X., Zhang Y., Kwong J.S., Zhang C., Li S., Sun F., Niu Y., Du L. (2015). The methodological quality assessment tools for preclinical and clinical studies, systematic review and meta-analysis, and clinical practice guideline: A systematic review. J. Evid. Based Med..

[24] Milman N. (2006). Iron and pregnancy—A delicate balance. Ann. Hematol..

[25] Ross A.C. (2017). Impact of chronic and acute inflammation on extra- and intracellular iron homeostasis. Am. J. Clin. Nutr..

[26] Higgins J.P., Thompson S.G. (2004). Controlling the risk of spurious findings from meta-regression. Stat. Med..

[27] Rios E., Lipschitz D.A., Cook J.D., Smith N.J. (1975). Relationship of maternal and infant iron stores as assessed by determination of plasma ferritin. Pediatrics.

[28] Murray M.J., Murray A.B., Murray N.J., Murray M.B. (1978). The effect of iron status of Nigerien mothers on that of their infants at birth and 6 months, and on the concentration of Fe in breast milk. Br. J. Nutr..

[29] Kilbride J., Baker T.G., Parapia L.A., Khoury S.A. (2000). Iron status, serum folate and B12 values in pregnancy and postpartum: Report from a study in Jordan. Ann. Saudi. Med..

[30] Poyrazoǧlu H.G., Denizmen Aygün A., Üstündaǧ B., Akarsu S., Yildirmaz S. (2011). Iron status of pregnant women and their newborns, and the necessity of iron supplementation in infants in eastern Turkey. Turk. Pediatr. Ars..

[31] Hanieh S., Ha T.T., Simpson J.A., Casey G.C., Thuy T., Khuong N.C., Thoang D.D., Thuy D.D., Pasricha S.-R., Tran T.D. (2013). The effect of intermittent antenatal iron supplementation on infant outcomes in rural vietnam: A cluster randomised trial. Annals of nutrition & metabolism. J. Conf. Abstr..

[32] Kulik-Rechberger B., Kościesza A., Szponar E., Domosud J. (2016). Hepcidin and iron status in pregnant women and full-term newborns in first days of life. Ginekol. Pol..

[33] Matias S.L., Mridha M.K., Young R.T., Hussain S., Dewey K.G. (2018). Daily Maternal Lipid-Based Nutrient Supplementation with 20 mg Iron, Compared with Iron and Folic Acid with 60 mg Iron, Resulted in Lower Iron Status in Late Pregnancy but Not at 6 Months Postpartum in Either the Mothers or Their Infants in Bangladesh. J. Nutr..

[34] Puolakka J., Jänne O., Vihko R. (1980). Evaluation by serum ferritin assay of the influence of maternal iron stores on the iron status of newborns and infants. Acta Obstet. Gynecol. Scand. Suppl..

[35] Milman N., Ibsen K.K., Christensen J.M. (1987). Serum ferritin and iron status in mothers and newborn infants. Acta Obstet. Gynecol. Scand..

[36] Morton R.E., Nysenbaum A., Price K. (1988). Iron status in the first year of life. J. Pediatr. Gastroenterol. Nutr..

[37] Preziosi P., Prual A., Galan P., Daouda H., Boureima H., Hercberg S. (1997). Effect of iron supplementation on the iron status of pregnant women: Consequences for newborns. Am. J. Clin. Nutr..

[38] Liu L., Xiao Y., Zou B., Zhao L.L. (2015). Study of the significance of iron deficiency indexes and erythrocyte parameters in anemic pregnant women and their newborns. Genet. Mol. Res..

[39] Santos D.C.C., Angulo-Barroso R.M., Li M., Bian Y., Sturza J., Richards B., Lozoff B. (2018). Timing, duration, and severity of iron deficiency in early development and motor outcomes at 9 months. Eur. J. Clin. Nutr..

[40] Abioye A.I., McDonald E.A., Park S., Ripp K., Bennett B., Wu H.W., Pond-Tor S., Sagliba M.J., Amoylen A.J., Baltazar P.I. (2019). Maternal anemia type during pregnancy is associated with anemia risk among offspring during infancy. Pediatr. Res..

[41] Shukla A.K., Srivastava S., Verma G. (2019). Effect of maternal anemia on the status of iron stores in infants: A cohort study. J. Family Community Med..

[42] Turkay S., Tanzer F., Gultekin A., Bakici M.Z. (1995). The influence of maternal iron deficiency anaemia on the haemoglobin concentration of the infant. J. Trop. Pediatr..

[43] Zhou S.J., Gibson R.A., Crowther C.A., Baghurst P., Makrides M. (2006). Effect of iron supplementation during pregnancy on the intelligence quotient and behavior of children at 4 y of age: Long-term follow-up of a randomized controlled trial. Am. J. Clin. Nutr..

[44] Davidson P.W., Strain J.J., Myers G.J., Thurston S.W., Bonham M.P., Shamlaye C.F., Stokes-Riner A., Wallace J.M.W., Robson P.J., Duffy E.M. (2008). Neurodevelopmental effects of maternal nutritional status and exposure to methylmercury from eating fish during pregnancy. Neurotoxicology.

[45] Rioux F.M., Bélanger-Plourde J., Leblanc C.P., Vigneau F. (2011). Relationship between maternal DHA and iron status and infants’ cognitive performance. Can. J. Diet. Pract. Res..

[46] Lewis S.J., Bonilla C., Brion M.J., Lawlor D.A., Gunnell D., Ben-Shlomo Y., Ness A., Smith G.D. (2014). Maternal iron levels early in pregnancy are not associated with offspring IQ score at age 8, findings from a Mendelian randomization study. Eur. J. Clin. Nutr..

[47] Tran T.D., Tran T., Simpson J.A., Tran H.T., Nguyen T.T., Hanieh S., Dwyer T., Biggs B.-A., Fisher J. (2014). Infant motor development in rural Vietnam and intrauterine exposures to anaemia, iron deficiency and common mental disorders: A prospective community-based study. BMC Pregnancy Childbirth.

[48] Lou J., Mai X., Lozoff B., Kileny P.R., Felt B.T., Zhao Z., Shao J. (2015). Prenatal iron deficiency and auditory brainstem responses at 3 and 10 months: A pilot study. Hong Kong J. Paediatr..

[49] Mireku M.O., Davidson L.L., Boivin M.J., Zoumenou R., Massougbodji A., Cot M., Bodeau-Livinec F. (2016). Prenatal iron deficiency, neonatal ferritin, and infant cognitive function. Pediatrics.

[50] Vaughn J., Brown J., Carter J.P. (1986). The effects of maternal anemia on infant behavior. J. Natl. Med. Assoc..

[51] Hernández-Martínez C., Canals J., Aranda N., Ribot B., Escribano J., Arija V. (2011). Effects of iron deficiency on neonatal behavior at different stages of pregnancy. Early Hum. Dev..

[52] Tran T.D., Biggs B.A., Tran T., Simpson J.A., Hanieh S., Dwyer T., Fisher J. (2013). Impact on Infants’ Cognitive Development of Antenatal Exposure to Iron Deficiency Disorder and Common Mental Disorders. PLoS ONE.

[53] Koubaa S., Hällström T., Brismar K., Hellström P.M., Hirschberg A.L. (2015). Biomarkers of nutrition and stress in pregnant women with a history of eating disorders in relation to head circumference and neurocognitive function of the offspring. BMC Pregnancy Childbirth.

[54] Park S., Bellinger D.C., Adamo M., Bennett B., Choi N.K., Baltazar P.I., Ayaso E.B., Monterde D.B.S., Tallo V., Olveda R.M. (2016). Mechanistic pathways from early gestation through infancy and neurodevelopment. Pediatrics.

[55] Berglund S.K., Torres-Espínola F.J., García-Valdés L., Segura M.T., Martínez-Zaldívar C., Padilla C., Rueda R., García M.P., McArdle H.J., Campoy C. (2017). The impacts of maternal iron deficiency and being overweight during pregnancy on neurodevelopment of the offspring. Br. J. Nutr..

[56] Choudhury V., Amin S.B., Agarwal A., Srivastava L., Soni A., Saluja S. (2015). Latent iron deficiency at birth influences auditory neural maturation in late preterm and term infants. Am. J. Clin. Nutr..

[57] ElAlfy M.S., Ali El-Farrash R., Mohammed T.H., Abdel Rahman Ismail E., Ahmed Mokhtar N. (2018). Auditory brainstem response in full-term neonates born to mothers with iron deficiency anemia: Relation to disease severity. J. Matern. Fetal Neonatal Med..

[58] Arija V., Hernández-Martínez C., Tous M., Canals J., Guxens M., Fernández-Barrés S., Ibarluzea J., Babarro I., Soler-Blasco R., Llop S. (2019). Association of iron status and intake during pregnancy with neuropsychological outcomes in children aged 7 years: The prospective birth cohort infancia y medio ambiente (INMA) study. Nutrients.

[59] Kupsco A., Estrada-Gutierrez G., Cantoral A., Schnaas L., Pantic I., Amarasiriwardena C., Svensson K., Bellinger D.C., Téllez-Rojo M.M., Baccarelli A.A. (2020). Modification of the effects of prenatal manganese exposure on child neurodevelopment by maternal anemia and iron deficiency. Pediatr. Res..

[60] Santa-Marina L., Lertxundi N., Andiarena A., Irizar A., Sunyer J., Molinuevo A., Llop S., Julvez J., Beneito A., Ibarluzea J. (2020). Maternal ferritin levels during pregnancy and ADHD symptoms in 4-year-old children: Results from the INMA–infancia y medio ambiente (environment and childhood) prospective birth cohort study. Int. J. Environ. Res. Public Health.

[61] Ganpule A., Yajnik C.S., Fall C.H.D., Rao S., Fisher D.J., Kanade A., Cooper C., Naik S., Joshi N., Lubree H. (2006). Bone mass in Indian children—Relationships to maternal nutritional status and diet during pregnancy: The Pune maternal nutrition study. J. Clin. Endocrinol. Metab..

[62] Alwan N.A., Lawlor D.A., McArdle H.J., Greenwood D.C., Cade J.E. (2012). Exploring the relationship between maternal iron status and offspring’s blood pressure and adiposity: A Mendelian randomization Study. Clin. Epidemiol..

[63] Alwan N.A., Cade J.E., McArdle H.J., Greenwood D.C., Hayes H.E., Ciantar E., Simpson N.A.B. (2015). Infant arterial stiffness and maternal iron status in pregnancy: A UK birth cohort (Baby VIP Study). Neonatology.

[64] Nwaru B.I., Hayes H., Gambling L., Craig L.C.A., Allan K., Prabhu N., Turner S.W., McNeill G., Erkkola M., Seaton A. (2014). An exploratory study of the associations between maternal iron status in pregnancy and childhood wheeze and atopy. Br. J. Nutr..

[65] Bédard A., Lewis S.J., Burgess S., Henderson A.J., Shaheen S.O. (2018). Maternal iron status during pregnancy and respiratory and atopic outcomes in the offspring: A Mendelian randomisation study. BMJ Open Respir. Res..

[66] Abioye A.I., Aboud S., Premji Z., Etheredge A.J., Gunaratna N.S., Sudfeld C.R., Mongi R., Meloney L., Darling A.M., Noor R.A. (2016). Iron supplementation affects hematologic biomarker concentrations and pregnancy outcomes among iron-deficient tanzanian women1-3. J. Nutr..

[67] Hanieh S., Ha T.T., De Livera A.M., Simpson J.A., Thuy T.T., Khuong N.C., Thoang D.D., Tran T.D., Tuan T., Fisher J. (2015). Antenatal and early infant predictors of postnatal growth in rural Vietnam: A prospective cohort study. Arch. Dis. Child..

[68] Goldenberg R.L., Mercer B.M., Miodovnik M., Thurnau G.R., Meis P.J., Moawad A., Paul R.H., Bottoms S.F., Das A., Roberts J.M. (1998). Plasma ferritin, premature rupture of membranes, and pregnancy outcome. Am. J. Obstet. Gynecol..

[69] Stordal K., McArdle H.J., Hayes H., Tapia G., Viken M.K., Lund-Blix N.A., Haugen M., Joner G., Skrivarhaug T., Mårild K. (2018). Prenatal iron exposure and childhood type 1 diabetes. Sci. Rep..

[70] Dai A.I., Demiryürek S., Aksoy S.N., Perk P., Saygili O., Güngör K. (2015). Maternal iron deficiency anemia as a risk factor for the development of retinopathy of prematurity. Pediatr. Neurol..

[71] Balesaria S., Hanif R., Salama M.F., Raja K., Bayele H.K., McArdle H., Srai S.K. (2012). Fetal iron levels are regulated by maternal and fetal Hfe genotype and dietary iron. Haematologica.

[72] Jaime-Perez J.C., Herrera-Garza J.L., Gomez-Almaguer D. (2005). Sub-Optimal Fetal Iron Acquisition under a Maternal Environment. Arch. Med. Res..

[73] Ilyes I., Jezerniczky J., Kovacs J., Dvoracsek E., Csorba S. (1985). Relationship of maternal and newborn (cord) serum ferritin concentrations measured by immunoradiometry. Acta. Paediatr. Hung.

[74] Allen L.H. (2000). Anemia and iron deficiency: Effects on pregnancy outcome. Am. J. Clin. Nutr..

[75] Scholl T.O. (2011). Maternal iron status: Relation to fetal growth, length of gestation, and iron endowment of the neonate. Nutr. Rev..

[76] Fisher A.L., Nemeth E. (2017). Iron homeostasis during pregnancy. Am. J. Clin. Nutr..

[77] Bothwell T.H. (2000). Iron requirements in pregnancy and strategies to meet them. Am. J. Clin. Nutr..

[78] Suchdev P.S., Williams A.M., Mei Z., Flores-Ayala R., Pasricha S.R., Rogers L.M., Namaste S.M. (2017). Assessment of iron status in settings of inflammation: Challenges and potential approaches. Am. J. Clin. Nutr..

[79] White K.C. (2005). Anemia is a poor predictor of iron deficiency among toddlers in the United States: For heme the bell tolls. Pediatrics.

[80] Beall C.M. (2020). Hemoglobin, altitude, and sensitive Swiss men. Blood.

[81] Gassmann M., Mairbäurl H., Livshits L., Seide S., Hackbusch M., Malczyk M., Kraut S., Gassmann N.N., Weissmann N., Muckenthaler M.U. (2019). The increase in hemoglobin concentration with altitude varies among human populations. Ann. NY Acad. Sci..

[82] Young M.F., Oaks B.M., Tandon S., Martorell R., Dewey K.G., Wendt A.S. (2019). Maternal hemoglobin concentrations across pregnancy and maternal and child health: A systematic review and meta-analysis. Ann. NY Acad. Sci..

