# Supplementary material for: Maternal Iron Status in Pregnancy and Child Health Outcomes after Birth: A Systematic Review and Meta-Analysis
Source: Nutrients. 2021 Jun 28;13(7):2221. doi: 10.3390/nu13072221 (PMC8308244; doi:10.3390/nu13072221)
Supplement: Supplementary file 1 [file nutrients-13-02221-s001.zip › nutrients-1255829-supplementary.pdf]

On-line Supplementary Material

Maternal Iron Status in Pregnancy and Child Health Outcomes after Birth: A Systematic Review and Meta-Analysis.

Short title: Maternal iron status in pregnancy and child health

Hugo G. Quezada-Pinedo [1,2]; Florian Cassel [3]; Liesbeth Duijts [3,4]; Martina U. Muckenthaler [5]; Max Gassmann [6,7]; Vincent W.V. Jaddoe [1,2]; Irwin K. M. Reiss [1,3]; Marijn J. Vermeulen [3].

[1]The Generation R Study Group, [2]Department of Pediatrics; [3]Department of Pediatrics, Division of Neonatology; [4]Department of Pediatrics, Division of Respiratory Medicine and Allergology, Erasmus MC, University Medical Center, Rotterdam, The Netherlands, [5]University Hospital Heidelberg, Germany; Molecular Medicine Partnership Unit, [6]Institute of Veterinary Physiology, Vetsuisse Faculty, and Zurich Center for Integrative, Human Physiology, University of Zurich, Zurich, Switzerland, [7]Universidad Peruana Cayetano Heredia, Lima, Peru

Contents

Supplementary methods 3
Supplementary Table 1. Estimated risk of bias of included studies 6
Supplementary Table 2. Meta-regression on studies on child haemoglobin or ferritin concentrations 8
Supplementary Figure 1. Funnel plots for studies on child ferritin and haemoglobin 9

Supplementary methods

Literature search terms

Embase

('iron blood level'/exp OR 'iron deficiency anemia'/exp OR 'iron overload'/exp OR 'iron deficiency'/exp OR 'hemochromatosis'/exp OR 'hemosiderosis'/exp OR 'siderosis'/exp OR 'hepcidin'/exp OR 'ferritin blood level'/exp OR 'hyperferritinemia'/exp OR 'transferrin blood level'/exp OR 'iron therapy'/exp OR 'iron intake'/exp OR (hemochromatos* OR haemochromatos* OR hemosideros* OR haemosideros* OR siderochromatos* OR hyperferr* OR hypoferr* OR hypersiderem* OR hyposiderem* OR siderem* OR sideros* OR sideropen* OR ((free*) NEXT/1 (iron* OR ferro* OR ferrum* OR Fe)) OR ((iron* OR ferro* OR ferrum* OR Fe OR Apoferritin* OR apotransferrin* OR transferrin OR transferrins* OR isotransferrin* OR serotransferrin* OR siderophilin* OR ferrichrome* OR ferritin* OR ferroportin* OR hemosiderin* OR hephaestin* OR isoferritin* OR hepcidin* OR LEAP-1 OR prohepcidin* OR pro-hepcidin*) NEAR/6 (accumulate* OR absorption* OR blood* OR plasma* OR serum* OR deficien* OR increas* OR decreas* OR high* OR low OR lower OR lowest* OR overload OR excess* OR intoxic* OR poison* OR toxic* OR binding-protein* OR depletion* OR status* OR state* OR level* OR storag* OR store* OR deposition* OR refractor* OR intake* OR supplement* OR diet* OR therapy OR therapies* OR treatment*)) OR ((hypochrom* OR microcytic* OR ferripriv* OR iron OR fe) NEAR/3 (anaemi* OR anemi*))):ab,ti,kw) AND ('pregnancy'/exp OR 'fetus'/exp OR 'prenatal exposure'/exp OR 'maternal plasma'/de OR 'maternal serum'/de OR (pregnan* OR childbear* OR gestation* OR gravidit* OR embryo* OR fetus* OR foetal* OR fetal* OR prenatal* OR perinatal* OR ((child*) NEAR/3 (bear*)) OR maternal*):ab,ti,kw) AND ('progeny'/exp OR 'cohort analysis'/exp OR 'longitudinal study'/de OR 'prospective study'/de OR 'retrospective study'/de OR 'birth weight'/exp OR (progen* OR descendent* OR offspring* OR off-spring* OR cohort* OR follow-up OR long-term OR longitudinal OR prospectiv* OR retrospectiv* OR child* OR mothers-with OR maternal* OR birthweight* OR neonatal* OR newborn*):ab,ti,kw) NOT ([animals]/lim NOT [humans]/lim) AND [english]/lim NOT ([Conference Abstract]/lim)

Medline

(exp Anemia, Iron-Deficiency/ OR exp Iron Overload/ OR exp Hemochromatosis/ OR exp Hemosiderosis/ OR exp Siderosis/ OR exp Hepcidins/ OR exp Iron, Dietary/ OR (hemochromatos* OR haemochromatos* OR hemosideros* OR haemosideros* OR siderochromatos* OR hyperferr* OR hypoferr* OR hypersiderem* OR hyposiderem* OR siderem* OR sideros* OR sideropen* OR ((free*) ADJ (iron* OR ferro* OR ferrum* OR Fe)) OR ((iron* OR ferro* OR ferrum* OR Fe OR Apoferritin* OR apotransferrin* OR transferrin OR transferrins* OR isotransferrin* OR serotransferrin* OR siderophilin* OR ferrichrome* OR ferritin* OR ferroportin* OR hemosiderin* OR hephaestin* OR isoferritin* OR hepcidin* OR LEAP-1 OR prohepcidin* OR pro-hepcidin*) ADJ6 (accumulate* OR absorption* OR blood* OR plasma* OR serum* OR deficien* OR increas* OR decreas* OR high* OR low OR lower OR lowest* OR overload OR excess* OR intoxic* OR poison* OR toxic* OR binding-protein* OR depletion* OR status* OR state* OR level* OR storag* OR store* OR deposition* OR refractor* OR intake* OR supplement* OR diet* OR therapy OR therapies* OR treatment*)) OR ((hypochrom* OR microcytic* OR ferripriv* OR iron OR fe) ADJ3 (anaemi* OR anemi*))).ab,ti,kf.) AND (exp Pregnancy/ OR exp Gravidity/ OR exp Fetus/ OR exp Prenatal Exposure Delayed Effects/

OR exp Maternal Serum Screening Tests/ OR (pregnan* OR childbear* OR gestation* OR gravidit* OR embryo* OR fetus* OR foetal* OR fetal* OR prenatal* OR perinatal* OR ((child*) ADJ3 (bear*)) OR maternal*).ab,ti,kf.) AND (exp Cohort Studies/ OR exp Birth Weight/ OR (progen* OR descendent* OR offspring* OR off-spring* OR cohort* OR follow-up OR long-term OR longitudinal OR prospectiv* OR retrospectiv* OR child* OR mothers-with OR maternal* OR birthweight* OR neonatal* OR newborn*).ab,ti,kf.) NOT (exp animals/ NOT humans/) AND english.la. NOT (news OR congres* OR abstract* OR book* OR chapter* OR dissertation abstract*).pt.

Cochrane Central

((hemochromatos* OR haemochromatos* OR hemosideros* OR haemosideros* OR siderochromatos* OR hyperferr* OR hypoferr* OR hypersiderem* OR hyposiderem* OR siderem* OR sideros* OR sideropen* OR ((free*) NEXT/1 (iron* OR ferro* OR ferrum* OR Fe)) OR ((iron* OR ferro* OR ferrum* OR Fe OR Apoferritin* OR apotransferrin* OR transferrin OR transferrins* OR isotransferrin* OR serotransferrin* OR siderophilin* OR ferrichrome* OR ferritin* OR ferroportin* OR hemosiderin* OR hephaestin* OR isoferritin* OR hepcidin* OR LEAP-1 OR prohepcidin* OR pro-hepcidin*) NEAR/6 (accumulate* OR absorption* OR blood* OR plasma* OR serum* OR deficien* OR increas* OR decreas* OR high* OR low OR lower OR lowest* OR overload OR excess* OR intoxic* OR poison* OR toxic* OR binding-protein* OR depletion* OR status* OR state* OR level* OR storag* OR store* OR deposition* OR refractor* OR intake* OR supplement* OR diet* OR therapy OR therapies* OR treatment*)) OR ((hypochrom* OR microcytic* OR ferripriv* OR iron OR fe) NEAR/3 (anaemi* OR anemi*))):ab,ti) AND ((pregnan* OR childbear* OR gestation* OR gravidit* OR embryo* OR fetus* OR foetal* OR fetal* OR prenatal* OR perinatal* OR ((child*) NEAR/3 (bear*)) OR maternal*):ab,ti) AND ((progen* OR descendent* OR offspring* OR ((off) NEXT/1 (spring*)) OR cohort* OR follow-up OR long-term OR longitudinal OR prospectiv* OR retrospectiv* OR child* OR mothers-with OR maternal* OR birthweight* OR neonatal* OR newborn*):ab,ti)

Web of Science

TS=(((hemochromatos* OR haemochromatos* OR hemosideros* OR haemosideros* OR siderochromatos* OR hyperferr* OR hypoferr* OR hypersiderem* OR hyposiderem* OR siderem* OR sideros* OR sideropen* OR ((free*) NEAR/1 (iron* OR ferro* OR ferrum* OR Fe)) OR ((iron* OR ferro* OR ferrum* OR Fe OR Apoferritin* OR apotransferrin* OR transferrin OR transferrins* OR isotransferrin* OR serotransferrin* OR siderophilin* OR ferrichrome* OR ferritin* OR ferroportin* OR hemosiderin* OR hephaestin* OR isoferritin* OR hepcidin* OR LEAP-1 OR prohepcidin* OR pro-hepcidin*) NEAR/5 (accumulate* OR absorption* OR blood* OR plasma* OR serum* OR deficien* OR increas* OR decreas* OR high* OR low OR lower OR lowest* OR overload OR excess* OR intoxic* OR poison* OR toxic* OR binding-protein* OR depletion* OR status* OR state* OR level* OR storag* OR store* OR deposition* OR refractor* OR intake* OR supplement* OR diet* OR therapy OR therapies* OR treatment*)) OR ((hypochrom* OR microcytic* OR ferripriv* OR iron OR fe) NEAR/2 (anaemi* OR anemi*)))) AND ((pregnan* OR childbear* OR gestation* OR gravidit* OR embryo* OR fetus* OR foetal* OR fetal* OR prenatal* OR perinatal* OR ((child*) NEAR/2 (bear*)) OR maternal*)) AND ((progen* OR descendent* OR offspring* OR off-spring* OR cohort* OR follow-up OR long-term OR longitudinal OR prospectiv* OR retrospectiv* OR child* OR mothers-with OR maternal* OR birthweight* OR neonatal* OR newborn*)) NOT ((animal* OR rat OR rats OR mouse OR mice OR murine OR dog OR dogs OR canine OR cat OR cats OR feline OR rabbit OR cow OR cows OR bovine

OR rodent* OR sheep OR ovine OR pig OR swine OR porcine OR veterinar* OR chick* OR zebrafish* OR baboon* OR nonhuman* OR primate* OR cattle* OR goose OR geese OR duck OR macaque* OR avian* OR bird* OR fish*) NOT (human* OR patient* OR women OR woman OR men OR man))) AND DT=(Article OR Review) AND LA=(English)

Google Scholar

"hemochromatosis|hemosiderosis|hyperferritinemia|siderosis"|"iron|fe|ferritin|transferrin|hepcidin level|status|anemia|overload|deficiency" "pregnancy|gravidity|fetus|prenatal|perinatal" "progeny|offspring"

Supplementary Table S1. Estimated risk of bias of included studies

Reference	Year	Country	Study design	Criteria								Total score	Risk of bias
				Selection				Comparability	Outcome				
				1	2	3	4	5	6	7	8		
Rios	1975	USA	Cohort			*	*			*	*	4	Medium
Murray	1978	Niger	Cohort		*	*	*			*		4	Medium
Puolakka	1980	Finland	Cohort			*	*			*		3	High
Vaughn	1986	USA	Cohort			*	*			*		3	High
Milman	1987	Denmark	Cohort		*	*	*			*		4	Medium
Morton	1988	UK	Cohort		*	*	*			*		4	Medium
Turkay	1995	Turkey	Cohort		*	*	*			*	*	5	Medium
Preziosi	1997	Niger	Clinical trial			*	*	*		*	*	5	Medium
Goldenberg	1998	USA	Cohort	*	*	*	*	*	*	*	*	8	Low
Kilbride	2000	Jordan	Case control	*			*				*	3	High
Zhou	2006	Australia	Clinical trial	*	*	*	*	*	*	*	*	8	Low
Ganpule	2006	India	Cohort	*	*	*	*	*	*	*	*	8	Low
Davidson	2008	Seychelles	Cohort	*	*	*	*	*	*	*	*	8	Low
Poyrazoğlu	2011	Turkey	Cohort		*	*	*		*	*		5	Medium
Hernández	2011	Spain	Cohort	*	*	*	*	**		*		7	Low
Rioux	2011	Canada	Cohort	*	*	*	*	*		*		6	Medium
Alwan	2012	UK	Cohort	*	*	*	*	**	*	*		8	Low
Tran	2013	Vietnam	Cohort	*	*	*	*	*		*	*	7	Low
Hanieh	2013	Vietnam	Clinical trial	*	*	*	*	*	*	*	*	8	Low
Tran	2014	Vietnam	Cohort	*	*	*	*	**		*	*	8	Low
Lewis	2014	UK	Cohort	*	*	*	*	**	*	*		8	Low
Nwaru	2014	UK	Cohort	*	*	*	*	*	*	*		7	Low
Liu	2015	China	Cohort	*	*	*	*	*		*	*	7	Low
Koubaa	2015	Sweden	Cohort	*	*	*	*		*	*	*	7	Low
Choudhury	2015	India	Cohort	*	*	*	*	*	*	*	*	8	Low
Lou	2015	China	Cohort	*	*	*	*	*	*	*	*	8	Low
Alwan	2015	UK	Cohort	*	*	*	*	*	*	*	*	8	Low
Hanieh	2015	Vietnam	Cohort	*	*	*	*	*	*	*	*	8	Low
Dai	2015	Turkey	Case control	*	*		*		*	*		5	Medium
Kulik	2016	Poland	Cohort		*	*	*			*	*	5	Medium
Mireku	2016	Benin	Cohort	*	*	*	*	**	*	*	*	9	Low

Reference	Year	Country	Study design	Criteria								Total score	Risk of bias
				Selection				Comparability	Outcome				
				1	2	3	4	5	6	7	8		
Park	2016	Philippines	Cohort	*	*	*	*	*	*	*	*	8	Low
Abioye	2016	Tanzania	Cohort	*	*	*	*		*	*	*	7	Low
Berglund	2017	Spain	Cohort	*	*	*	*	**	*	*	*	9	Low
Matias	2018	Bangladesh	Clinical trial	*	*	*	*	**	*	*		8	Low
Santos	2018	China	Clinical trial	*	*	*	*	*	*	*		7	Low
Elalfy	2018	Egypt	Case control	*	*		*	**	*	*	*	8	Low
Størdal	2018	Norway	Case control		*	*	*	**	*	*		7	Low
Bédard	2018	UK	Cohort	*	*	*	*	**	*	*	*	9	Low
Abioye	2019	Philippines	Nested cohort	*	*	*	*	**	*	*	*	9	Low
Shukla	2019	India	Cohort	*	*	*	*	*	*	*	*	8	Low
Arija	2019	Spain	Cohort	*	*	*	*	**	*	*	*	9	Low
Kupsco	2020	Mexico	Cohort	*	*	*	*	*	*	*	*	8	Low
Santa-Maria	2020	Spain	Cohort	*	*	*	*	*	*	*	*	8	Low

Risk of bias assessment by New Castle Ottawa Scale[19] in the studies (n=41). Criteria applied for Cohort studies and clinical trials: 1) Representativeness of the exposed cohort (exposure defined as abnormal iron status), 2) Selection of the non-exposed cohort (normal iron status), 3) Ascertainment of exposure, 4) Demonstration that outcome of interest was not present at start of study, 5) Comparability of cohorts on the basis of the design or analysis, 6) Assessment of outcome, 7) Was follow-up long enough for outcomes to occur, 8) Adequacy of follow up of cohorts. For case control: 1) Is the case definition adequate?, 2) Representativeness of the cases, 3) Selection of Controls, 4) Definition of Controls, 5) Comparability of cases and controls on the basis of the design or analysis, 6) Ascertainment of exposure, 7) Same method of ascertainment for cases and controls, 8) Non-Response rate. Studies were classified as low (7-9 points), medium (4-6 points) and high (1-3 points) risk of bias. *Represent 1 point.

Supplementary Table S2. Meta-regression on studies on child haemoglobin or ferritin concentrations

Variable	No. of studies	Estimate	95% confidence interval		P value	% Heterogeneity explained
**Child ferritin**						
**Bivariable analysis**						
Publication year	7	0.09	-1.02,	1.20	0.84	11
Country income[c]	7	10.96	3.00,	18.91	**0.02**	77
Sample size	7	0.02	-0.04,	0.08	0.48	25
Study design[a]	7	1.21	-22.72,	25.14	0.90	0
Supplementation[e]	7	-1.94	-35.06,	31.18	0.89	0
Risk of bias[b]	7	-0.06	-5.98,	5.85	0.98	0
Stage of pregnancy[d]	7	-12.37	-31.76,	7.02	0.16	17
Child age[f]	7	1.32	-3.95,	6.59	0.55	61
**Multivariable analysis**						
Publication year		-0.17	-1.96,	1.62	0.78	
Stage of pregnancy[d]	7	-16.38	-62.05,	29.29	0.34	53
Child age[f]		-0.53	-9.38,	8.32	0.86	
**Child hemoglobin**						
**Bivariable analysis**						
Publication year	12	0.01	-0.02,	0.04	0.43	0
Country income[c]	12	-0.21	-0.61,	0.19	0.27	2
Sample size	12	0.00	-0.00,	0.00	0.39	0
Study design[a]	12	0.32	-0.56,	1.19	0.44	0
Supplementation[e]	12	-0.65	-1.48,	0.17	0.11	0
Risk of bias[b]	12	0.06	-0.15,	0.27	0.55	0
Stage of pregnancy[d]	12	0.74	0.00,	1.48	0.05	0
Child age[f]	12	-0.13	-0.38,	0.12	0.27	0
**Multivariable analysis**						
Publication year		0.04	0.02,	0.05	**0.0006**	
Stage of pregnancy[d]	12	1.07	0.72,	1.42	**<0.0001**	96
Child age[f]		-0.26	-0.39,	-0.13	**0.0015**	

[a]Prospective cohort or not. [b]Base on Ottawa-Newcastle scale. [c]Country income according to World Bank classification (https://datahelpdesk.worldbank.org/knowledgebase/articles/906519). [d]Time at iron measurement during pregnancy classified as: first, second, third trimester and peri-partum. Bold values = P<0.05. [e]Yes or not. [f]Age of child at outcome assessment in months. Models were tested for robustness with a permutation test.

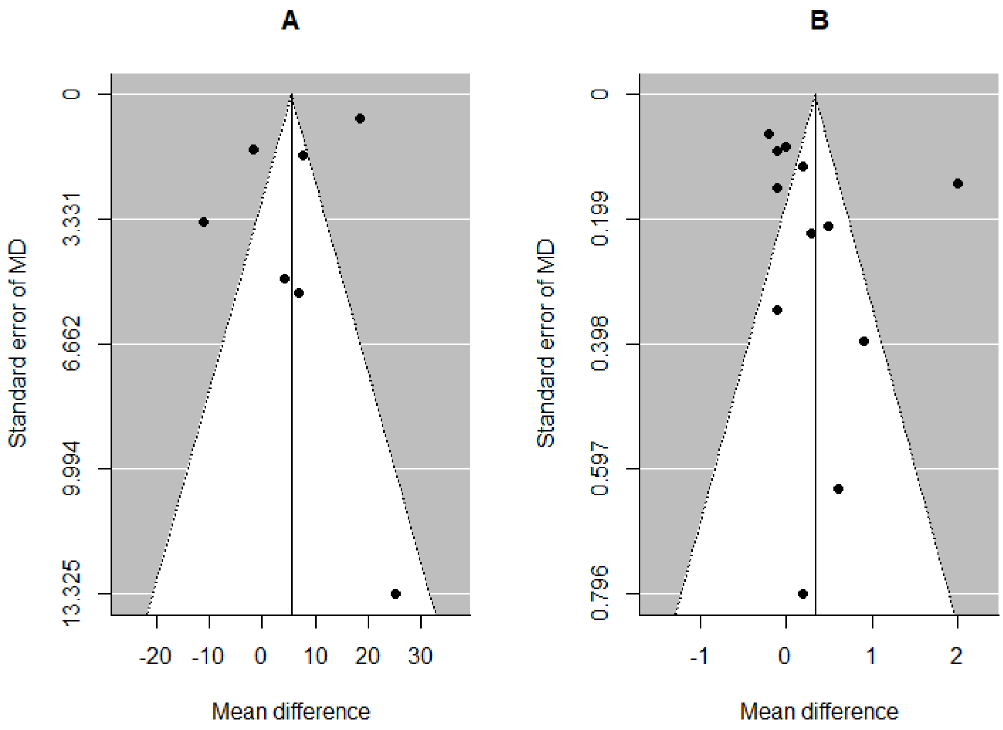

Supplementary Figure S1. Funnel plots for studies on child ferritin and haemoglobin

Funnel plot with 95% pseudo confidence limits of the studies included in the meta-analysis on the effects of maternal ferritin concentrations during pregnancy on (A) child ferritin concentrations, and (B) child hemoglobin concentrations. Egger's test for funnel plot asymmetry were T 0.52 (p=0.63), and 0.45 (p=0.66), respectively. MD, Mean difference.
